# Personal

**Published:** 1906-09

**Authors:** 


DR. SAINT CLAIR MCKELWAY, editor of the Brooklyn Eagle, is among the Americans of distinction now in England. Dr. McKelway is vice-chancellor of the University of the State of New York, and will represent that institution at the 400th Anniversary of Aberdeen University to be held about the middle of September.

Dr. C. W. Pillsbury, of Chicago, is spending a few weeks in Buffalo, calling upon physicians in the interest of the medical preparations of Armour and Company.

---

Dr. H. P. Sharp, of Arcade, N. Y., has been nominated for coroner of Wyoming County by the republican convention recently held in that county.

---

Dr. Eugene A. Smith, of Buffalo, who has been spending the summer in Europe, will return to his home and take up his professional work on or about September 1, 1906. During his absence Dr. Smith has visited the clinics of Mayo Robson, Moynihan, Lane, Cheyne, Hartman, Kocher and others. An interesting letter from him, in which he discusses the appendicitis problem, will be found elsewhere in this issue.

---

Dr. F. Park Lewis and family, of Buffalo, who have been spending the summer in touring through Europe, are expected to return about September 7, 1906.

---

Dr. H. S. Wende, of North Tonawanda, has been appointed veterinary surgeon to the fire department of that city for the ensuing year.

---

Dr. Herbert U. Williams, professor of pathology at the University of Buffalo, and who has been in Europe for the past few weeks, sailed from Hamburg, August 18, 1906, on the S. S. "Patricia." He expects to reach Buffalo about September 1.

---

Dr. Henry Schwarz, of Saint Louis, accompanied by his family, spent a few days in Buffalo and Niagara Falls, early in August. Dr. Hayd entertained them an afternoon at the baseball games.

---

Dr. and Mrs. Walter B. Dorsett, of Saint Louis, who visited Buffalo during the latter part of August, were entertained at dinner by Dr. H. E. Hayd of 493 Delaware Avenue, on Monday evening, August 20, 1906.

---

Dr. Edward Torrey, formerly of Allegany, N. Y., has removed to Olean where he has established his office at 136 N. First Street. Hours: 1 to 3, and 7 to 8, P. M. Telephone connections.

---

Dr. William S. Foster, of Pittsburg, was elected a vice-president of the American Medical Association, at its Annual Meeting held at Boston in June, 1906.

